# Prenatal hypoxia induces increased cardiac contractility on a background of decreased capillary density

**DOI:** 10.1186/1471-2261-9-1

**Published:** 2009-01-06

**Authors:** David Hauton, Victoria Ousley

**Affiliations:** 1 Department of Physiology, School of Clinical and Experimental Medicine, University of Birmingham, Birmingham, B15 2TT, UK; 2 Department of Vascular Studies, Northampton General Hospital, Northants, UK

## Abstract

**Background:**

Chronic hypoxia *in utero *(CHU) is one of the most common insults to fetal development and may be associated with poor cardiac recovery from ischaemia-reperfusion injury, yet the effects on normal cardiac mechanical performance are poorly understood.

**Methods:**

Pregnant female wistar rats were exposed to hypoxia (12% oxygen, balance nitrogen) for days 10–20 of pregnancy. Pups were born into normal room air and weaned normally. At 10 weeks of age, hearts were excised under anaesthesia and underwent retrograde 'Langendorff' perfusion. Mechanical performance was measured at constant filling pressure (100 cm H_2_O) with intraventricular balloon. Left ventricular free wall was dissected away and capillary density estimated following alkaline phosphatase staining. Expression of SERCA2a and Nitric Oxide Synthases (NOS) proteins were estimated by immunoblotting.

**Results:**

CHU significantly increased body mass (P < 0.001) compared with age-matched control rats but was without effect on relative cardiac mass. For incremental increases in left ventricular balloon volume, diastolic pressure was preserved. However, systolic pressure was significantly greater following CHU for balloon volume = 50 μl (P < 0.01) and up to 200 μl (P < 0.05). For higher balloon volumes systolic pressure was not significantly different from control. Developed pressures were correspondingly increased relative to controls for balloon volumes up to 250 μl (P < 0.05). Left ventricular free wall capillary density was significantly decreased in both epicardium (18%; P < 0.05) and endocardium (11%; P < 0.05) despite preserved coronary flow. Western blot analysis revealed no change to the expression of SERCA2a or nNOS but immuno-detectable eNOS protein was significantly decreased (P < 0.001) in cardiac tissue following chronic hypoxia *in utero*.

**Conclusion:**

These data offer potential mechanisms for poor recovery following ischaemia, including decreased coronary flow reserve and impaired angiogenesis with subsequent detrimental effects of post-natal cardiac performance.

## Background

Prenatal hypoxia may be one of the most common insults to the fetus during pregnancy. Exposures can vary from acute short-term insults [1] to chronic exposures (chronic hypoxia *in utero *– CHU) and have different impacts on fetal development. The impact of CHU on the heart and cardiac performance is unclear. The heart develops early during organogenesis and is functional at day 10 of gestation [2]. This coincides with the transition from a metabolic dependence upon glycolysis for ATP production (hence the absence of oxygen) and the transition to dependence on oxidative metabolism [3] and cardiac muscle fibre cross-sectional area showed a 5-fold increase between days 10–19 of embryo development in the rat [4]. This is indicative of significant hypertrophy resulting from the increased workload, and may be an indirect result of increased oxygenation. These changes indicate that the rat fetus may be relatively resistant to oxygen deprivation prior to this 10-day limit [5]. This is supported through studies of the Hypoxia-Inducible Factor (HIF-1α)-null mouse showing growth retardation at day 9 of development and death at day 10.5 [6], the abnormalities occurring in heart and blood vessels. Ultrasound studies of the human fetus reveal changes to regional blood flow during CHU [7], and dilatation of coronary vessels in growth-restricted human fetus [8].

CHU in lambs (initiated by high-altitude hypoxia) was without affect on capillary density, Capillary:Fibre (C:F) ratio and capillary volume density, but capillary diameter significantly increased [9]. Similar observations were noted following 20 days fetal anaemia in lambs (from day 115 gestation). However, in response to hypoxia a significant increase in rate of change of pressure development in myocardium (dP/dt max) was recorded [10], however this was not accompanied by changes to LV vessel number or size [10] but was compensated for by augmented coronary conductance measurements [11]. CHU in rats (12% oxygen – day 15–21 gestation) led to decreased recovery from ischemia-reperfusion injury in hearts following 25 min global ischaemia [12,13]. This was characterised by increased Caspase 3 activation and apoptosis [12]. Increases in left ventricle/body mass ratio and increased collagen I and III were also noted for CHU rats [13] suggesting adaptive cardiac remodelling.

For adult rats exposed to chronic hypoxia expression of endothelial Nitric Oxide Synthase (eNOS) was noted to increase at both mRNA and protein levels [14], however controversy remains over the impact of CHU on the expression of endothelial nitric oxide synthase (eNOS) in the developing fetus. No changes to eNOS expression in rat cardiac tissue were noted following CHU (12% oxygen – day 15–21 gestation) [13] however others report the absence of eNOS in cardiac tissue following CHU for days 15–21 of pregnancy [12] without compensatory increased expression of either iNOS or nNOS. By contrast, fetal hypoxia in near term guinea-pigs for 14 days between days 46–60 (term = 65 days) revealed preserved sensitivity to exogenous NO donors and an increase in eNOS mRNA expression [15]. More detailed analysis revealed preserved eNOS mRNA in coronary vessels, but decreased eNOS mRNA and protein in cardiomyocytes [16]. This may highlight important species differences in regulation of coronary flow, namely the importance of NO in the preservation of flow for the guinea pig [17-19] compared with the relative insensitivity of rat coronary flow to NOS inhibition [20,21]. Before investigating the impact of CHU on eNOS in the rat we must detail potential changes to cardiac work and factors that may influence coronary flow, including capillary density.

We hypothesise that exposure to CHU during this critical period when oxygen use increases (Day 10–20 of pregnancy) will increase neonatal cardiac work following catecholamine release and increasing coronary perfusion. The resulting high flow hypoxia will increase conductance and will reduce stretch-mediated angiogenic stimuli (as a result of shortened diastolic period) [22] resulting in decreased left ventricular capillary density.

Using the langendorff-perfused rat heart we will quantify the mechanical performance and measure basal coronary flow for adult rats subjected to CHU. In addition, we will estimate the protein expression of both calcium-handling proteins and the nitric oxide synthases to characterise the myocardium produced following exposure to CHU.

## Methods

### Materials

Oxygen-free nitrogen gas was obtained from British Oxygen Corporation (BOC, UK). Buffer reagents and nitro-blue tetrazolium reagent for determining capillary density were obtained from Sigma (Sigma Ltd, Poole, UK) and were of analytical grade or better. Massons Trichrome Stain to quantify tissue collagen was purchased from R.A. Lamb Ltd (Eastbourne, East Sussex, UK). Ventricular balloons were constructed 'in house' using Saran Wrap polythene film.

### Animals

Animals were maintained in accordance with the UK Home Office, Animal Scientific Procedures Act (1986) and housed at 22°C 12 hr light/12 hr dark with *ad libitum *access to food and water. After confirmation of mating, female Wistar rats (200 gm – Charles River, UK) were then housed singly in a hypoxic chamber and breathing room air. 10 days following mating the animals were exposed to a normobaric hypoxic atmosphere (12% oxygen, balance nitrogen) for 10 days. During this time the enclosed atmosphere was circulated through silica gel and soda lime to trap both water vapour and carbon dioxide. At 20 days post-mating the animals were transferred to room air and the pregnancy progressed as normal.

At birth, pups remained with the mother and were weaned normally. Following weaning at 4 weeks of age, the animals were divided by sex and the males housed with litter mates in groups (n = 5). These litters were then maintained for a further 10 weeks in normal room air with *ad libitum *access to both food and water.

### Surgical preparation

All experiments were carried out on male rats (mean age 11 weeks). Animals were prepared for surgery as outlined previously [23]. Briefly, animals were anaesthetised using halothane by inhalation (3.5% v/v in oxygen). The trachea was exposed and cannulated with a rigid tube for attachment of the spirometer (AD Instruments, Charlgrove, Oxfordshire, UK). Right femoral artery was cannulated for the estimation of arterial blood pressure and collection of blood samples. Patency of all cannulae was maintained by periodic flushing with phosphate-buffered saline containing bovine albumin (2% w/v) and trisodium citrate (20 mM final concentration). Arterial blood pressure was measured through a plastic catheter and connected to a pressure transducer (MEMSCAP, Skoppum, Norway). Heart rate and developed pressure were derived from this pressure recording.

### Tissue isolation and heart perfusion

Animals were prepared surgically as outlined previously [24]. Briefly, anaesthesia was induced with pentobarbital (60 mg/kg ip in saline) and following thoracotomy hearts excised with lungs and thymus *in situ *and immersed in ice-cold Krebs-Hensleit medium. Excess tissue was dissected away, the thymus divided to reveal the aortic arch and the aorta was trimmed at the level of the carotid artery branches and cannulated (16 G needle). Hearts were perfused in retrograde fashion as outlined previously [12,13,24]. Flow through the heart was established and extra tissue was dissected away. An incision was made in the right ventricle and the left atrial appendage removed. A small flexible non-elastic balloon was inserted into the left atrium through the mitral valve and into the left ventricle. This fluid-filled balloon was attached to a fine plastic catheter and connected to a pressure transducer (MEMSCAP, Skoppum, Norway) and a graduated syringe (0–1000 μl: Hamilton, Nevada, USA). Hearts were maintained at 37°C and perfused at a constant pressure (100 cm H_2_O) with a Krebs-Hensleit crystalloid medium supplemented with glucose (10 mM) and CaCl_2 _(1.3 mM) gassed with oxygen/CO_2 _(95:5). Developed pressure was measured following isovolumic contraction of the fluid-filled balloon and recorded to computer using a digital interface (AD Instruments, Chalgrove, Oxford, UK).

### Ventricular Performance

Ventricular performance was estimated as outlined previously [24]. Briefly, the initial balloon volume was adjusted until the diastolic pressure recorded measured 0 mmHg and the developed pressure (difference between systolic and diastolic pressures) was <10 mmHg. This was referred to as a balloon volume of Zero. Balloon volume was increased in incremental steps (50 μl) and developed pressure was recorded in real time. Pressures were allowed to stabilise until diastolic pressure remained constant before initiating further increases in balloon volume. Incremental increases in balloon volume were performed until the peak systolic pressure developed exceeded 200 mmHg. The balloon was then deflated and the process repeated. Coronary flow was estimated from timed collections of a known volume of perfusate and expressed as volume/unit time/unit mass of cardiac tissue. Typically, 3 ml of coronary effluent was collected with collection time measured and subsequently expressed relative to cardiac mass.

Ventricular performance was calculated off-line following the experiment using computer analysis software (Chart Version 5.0, AD Instruments, Chalgrove, Oxford, UK). Heart rate, systolic pressure, diastolic pressure and hence developed pressure were measured. Rate of change of pressure (+dP/dt) was calculated from the maxima of first order derivative of pressure trace. Rate pressure product (RPP) was calculated at each balloon volume as the product of heart rate (bpm) × developed pressure (mmHg).

### Capillary Density

Tissues were blotted dry and weighed. The atria and connective tissue were dissected away and discarded. A transverse midline incision across the left ventricle was made. The lower portion (apex) of the heart was taken and the left ventricular free wall dissected away and mounted onto cork disks (22 mm: R.A. Lamb, Eastbourne, East Sussex, UK) in Tissue-Tek OCT compound (Sakura, Torrance, CA) before freezing in liquid nitrogen-cooled isopentane. For selected hearts whole myocardium was snap frozen in liquid nitrogen for Western blotting. Cryostat sections (10 μm) were cut and fixed onto glass slides. Capillaries were visualised using an alkaline phosphatase method [25] using nitro-blue tetrazolium reagent generating an insoluble formazan pigment. Capillary density was quantified from digital images (magnification ×200) by estimating capillary number in transverse-sectioned regions of LV free wall of known area. Three fields of epicardium and endocardium from six non-consecutive sections (minimum separation 50 μm between sections) were counted. Care was taken to estimate capillary numbers in different regions of LV free wall for separate sections. Sections of endocardium and epicardium were counted at random and the viewer was blinded to the origins of the tissue. Data is expressed as capillary number/mm^2 ^cross-sectional area of LV myocardium. Selected tissue sections were stained with Masson's Trichrome to quantify collagen infiltration and fibrosis.

### Diffusion distance

Diffusion distance was calculated using the estimations of capillary density using the following equation R = 1000√Nπ where N is the number of capillaries/mm^2^. This value is equal to the mean half distance between two capillaries in cross section and represents an index of average capillary supply [26].

### Immunoblotting for proteins

Western blot analysis was carried out on frozen whole heart tissue following removal of adipose tissue and atria. Standard Western immunoblotting techniques were used for the detection and estimation of relative amounts of SERCA2, eNOS, iNOS and nNOS (20–80 μg protein). Briefly, cardiac tissue (50 mg) was powdered in liquid nitrogen and extracted with RIPA buffer containing protease inhibitors, before centrifugation (10,000 rpm for 10 min) and recovery of the supernatant. The PVDF-membranes were probed with antibodies specific for SERCA2 (Santa Cruz – dilution 1:1000), mouse monoclonal anti-eNOS (1–500 dilution), mouse monoclonal anti-iNOS (1–500 dilution), mouse monoclonal anti-nNOS (1–1000 dilution) (Becton Dickinson, Oxford, UK). Appropriate HRP-linked secondary antibodies were used and membranes developed using enhanced chemiluminesence (ECL) detection (Roche). Densitometry of Western blots was estimated using ImageJ software (NIH). Protein expression was corrected for the expression of an internal control (tubulin).

### Statistical analysis

Statistical analysis was carried out using Single Factor ANOVA analysis with Bonferroni correction for multiple comparisons where appropriate. Data represents mean ± standard deviation.

## Results

Prenatal hypoxia did not alter pregnancy rate or the numbers of pups born. Pregnancies were of normal duration and the pups born alive. Mean litter size for CHU rats was 9 pups (range 2–15) and for control rats mean litter size was 11 pups (range 2–16). Body mass of the pups at birth was not measured to avoid disturbing the mothers. Body mass estimates at weaning revealed that CHU rats were significantly heavier than controls (68 ± 5 gm control *vs *86 ± 8 gm CHU: P < 0.01).

### Post-mortem measurements

**Figure 1:**
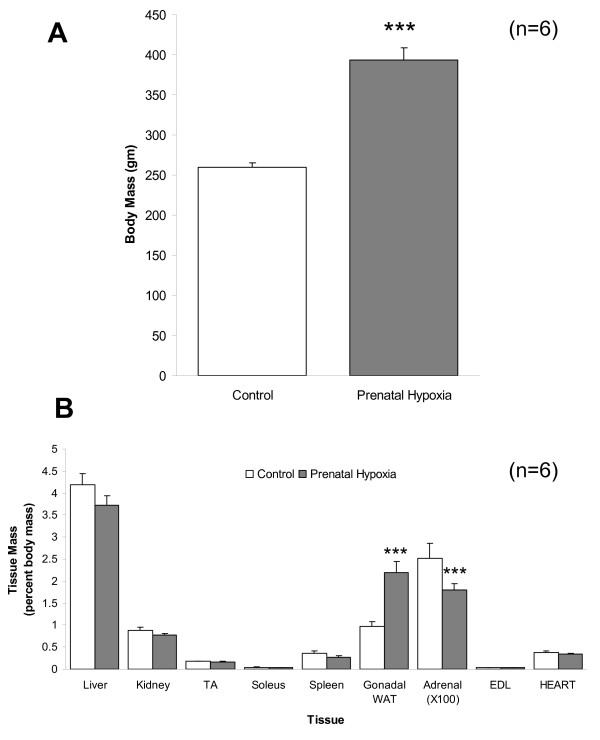
**Body and tissue mass at post-mortem**. Data represents body (A) and relative tissue masses (B) for control and adult offspring from pregnant female rats exposed to prenatal hypoxia. Tissue masses represented as a percentage of body mass to normalise for differences in body mass between control and CHU rats. Data represents Mean ± standard deviation (n = 6 for both control and CHU rats). Statistical comparisons represent: *** P < 0.001.

At post-mortem, body mass of CHU rats was significantly greater than age-matched controls (P < 0.001: Figure 1A). Absolute gonadal fat mass was significantly increased in CHU rats (2.25 ± 0.25 gm control *vs *8.37 ± 1.07 gm CHU; P < 0.001). Estimation of tissue mass as percentage of body mass revealed that gonadal white adipose tissue (WAT) increased ~2-fold in the CHU rats compared with controls (P < 0.001; Figure 1B) whereas Adrenal mass for the CHU rats decreased ~30% compared with controls (P < 0.001; Figure 1B), although no differences were seen in absolute adrenal mass (65 ± 9 mg control *vs *70 ± 5 mg CHU). Absolute cardiac mass for CHU rats was significantly greater than for controls (0.97 ± 0.1 gm control *vs *1.33 ± 0.07 gm CHU; P < 0.001) however, relative heart mass in CHU rats was not significantly different from control rats (Figure 1B).

Direct measurement of *in vivo *mean arterial blood pressure (MAP) revealed a 10% increase in MAP for anaesthetised CHU rats when compared with control, however this did not reach statistical significance (Table 1): in addition, heart rate was unchanged between control and CHU rats.

**Table 1:** Blood pressure and heart rate under anaesthesia and at post-mortem for control rats and adult offspring from pregnant female rats exposed to hypoxia during pregnancy.

**Measurement**	**Number**	**Control**	**Prenatally Hypoxic**	**Statistical Comparison**
*In vivo *Heart Rate (beats.min^-1^)	5	405 ± 60	397 ± 55	NS
Mean arterial blood pressure (mmHg)	5	115 ± 15	126 ± 15	NS
				
*In vitro *Heart Rate (beats.min^-1^)	10	277 ± 33	251.8 ± 24.3	NS
Estimated End Diastolic Volume (μl)	10	292.1 ± 44.7	304.9 ± 32.0	NS

Data represents Mean ± SD for control and CHU rats. Statistical comparisons are using a single factor ANOVA with statistical significance set at P < 0.05.

*Ex vivo *heart rate was measured when stable perfusion was established, and no difference was noted between hearts from control and CHU rats (table 1). End-diastolic volume (calculated from the linear regression of diastolic performance curve for values greater than zero at the point the regression line bisected the balloon volume at diastolic pressure = Zero) was increased by 4% in CHU rats (292 ± 45 μl control *vs *305 ± 32 μl CHU), (NS: Table 1).

### *Ex vivo *cardiac performance

**Figure 2:**
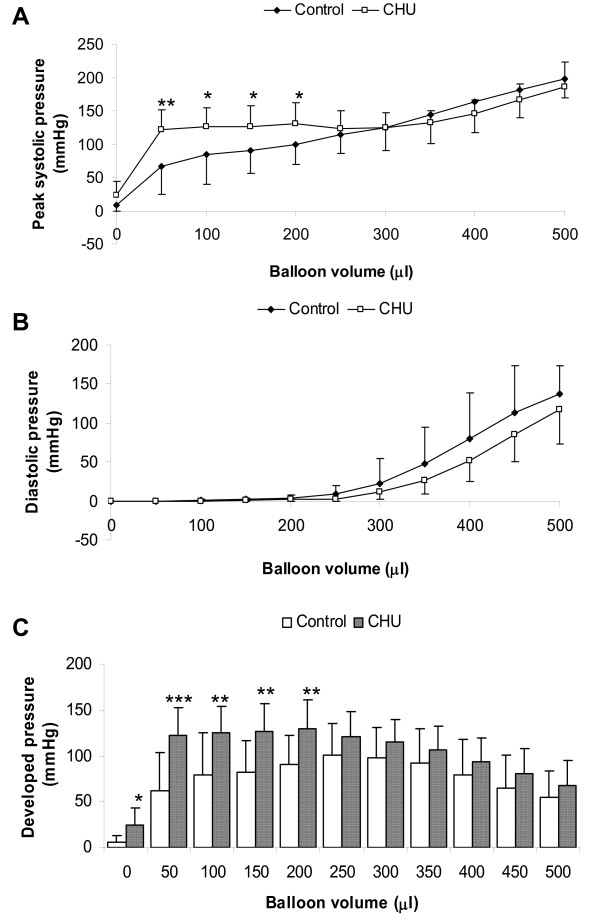
**Cardiac performance**. Data represents measurements taken from Langendorff-perfused hearts for control rats and adult offspring from pregnant female rats exposed to prenatal hypoxia (A) Peak systolic pressure (B) Diastolic pressure (C) Developed pressure. Data represents Mean ± standard deviation (n = 10 hearts). Statistical comparisons represent: * P < 0.05; ** P < 0.01; ***P < 0.001.

With increasing ventricular balloon volume the peak systolic pressure increased for control animals (Figure 2A). For CHU rats peak systolic pressure reached an initial peak of 125 mmHg at balloon volume of 50 μl (P < 0.01) and the developed pressure then remained at this plateau value (~125 mmHg) with increasing balloon volume up to balloon volume = 400 μl. Peak systolic pressure measured for CHU rats was significantly greater than control for increasing balloon volumes up to 200 μl (P < 0.05) at which point the peak systolic pressure followed the same pattern as control rats (Figure 2A). Measured diastolic pressure was not significantly different between control and CHU rats with increasing balloon volume (figure 2B). LV developed pressure per unit cardiac mass increased with increasing balloon volume for control rats, reaching a peak of 100 mmHg at balloon volume = 250 μl. For CHU rats developed pressure peaked at 120 mmHg at balloon volume = 50 μl, 2-fold higher than that recorded for controls at an equivalent balloon volume (P < 0.001) (Figure 2C). The developed pressure remained constant up to 250 μl balloon volume (P < 0.05, compared with control) and continued to decline in a pattern similar to control rats (Figure 2C) with subsequent increases in balloon volume. For balloon volumes >250 μl, developed pressures generated for CHU heart LV were not significantly different from control hearts.

**Figure 3:**
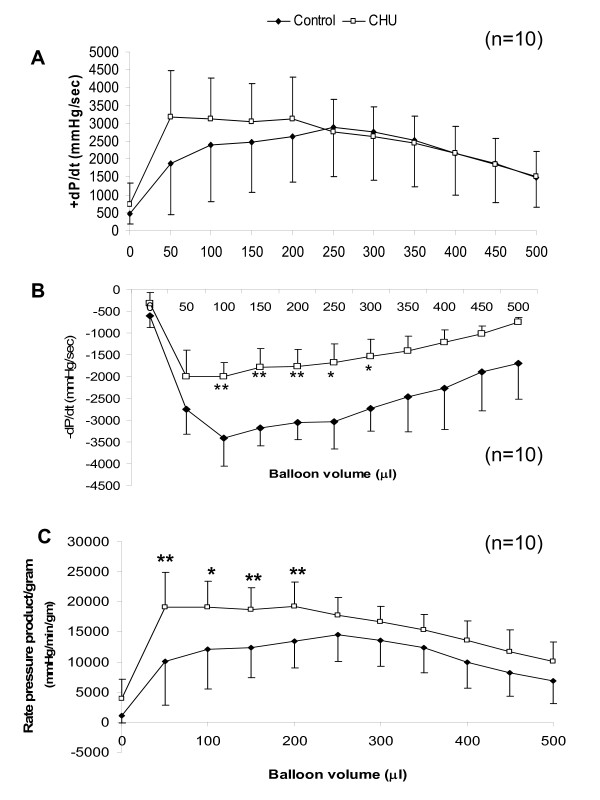
**Cardiac contractility and cardiac work**. (A). Peak +dP/dt (B) Peak -dP/dt (C). Rate-pressure product Data represents Mean ± standard deviation (n = 10 hearts). Statistical comparisons represent: * P < 0.05; ** P < 0.01.

Rate of pressure development (measured as +dP/dt) reached a maximum in control rats at balloon volume = 250 μl and subsequently declined (figure 3A). For CHU rats dP/dt reached a peak at 50 μl and was sustained until balloon volume = 200 μl, declining thereafter in a pattern corresponding to that of control rats (Figure 3A). Rate of ventricular relaxation (measured as -dP/dt) was decreased at all points when compared with control hearts (figure 3B) reaching statistical significance at balloon volumes >50 μl (P < 0.01). Maximum -dP/dt was achieved at low balloon volume (100 μl) for controls and rate decreased with increasing balloon volume (figure 3B). Rate-pressure product (RPP) expressed per unit cardiac mass followed a similar pattern to dP/dt in control rats, peaking at balloon volume = 250 μl (Figure 3C), whereas for CHU rats RPP reached a maxima at balloon volume = 50 μl, (P < 0.01; Figure 3C). RPP was maintained at this peak value for increasing balloon volumes up to a maximum of 200 μl for CHU rats (P < 0.01 *vs *control). For further increases in balloon volume RPP declined in a similar pattern to controls (Figure 3C).

### Coronary flow and capillary density

**Figure 4:**
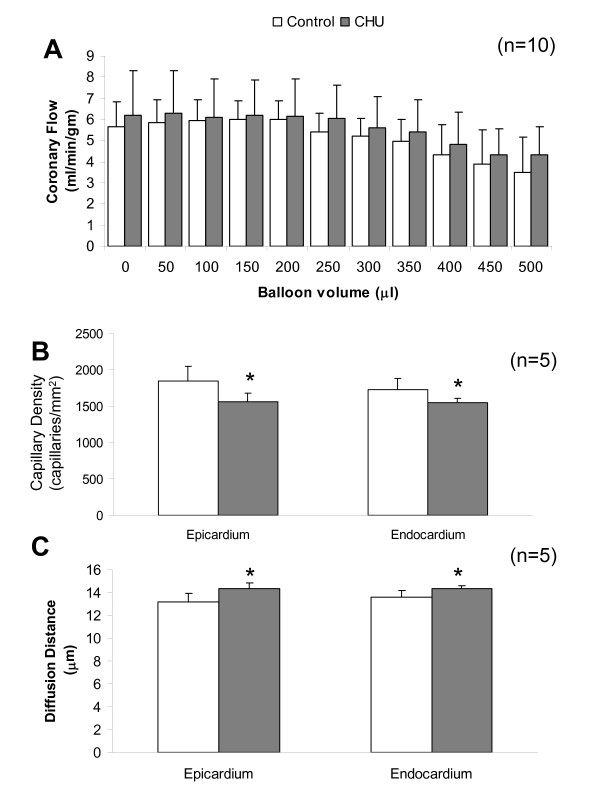
**Coronary flow, capillary density and oxygen diffusion**. (A) Coronary flow was measured as timed recovery of a known volume of perfusate from coronary drainage. Data represents Mean ± standard deviation (n = 10 hearts). (B) Capillary density. (C) Oxygen diffusion distance. Data represents Mean ± standard deviation (n = 5 hearts). Statistical comparisons represent: * P < 0.05.

Coronary flow decreased with increasing balloon volume similarly for control rats and CHU rats (NS; Figure 4A). There was a 7% decrease in capillary density between epicardium and endocardium for control rats, (NS; Figure 4B). A 19% lower capillary density (P < 0.05) was recorded for epicardium in CHU rats when compared with control rats, with endocardium showing an 11% lower capillary density (P < 0.05) (Figure 4B). The calculated oxygen diffusion distance for epicardium and endocardium was significantly increased in CHU rats (P < 0.05 for both; Figure 4C). Estimation of percentage volume density of collagen recorded no difference in collagen infiltration between control and CHU hearts (data not shown).

### Western blot analysis

**Figure 5:**
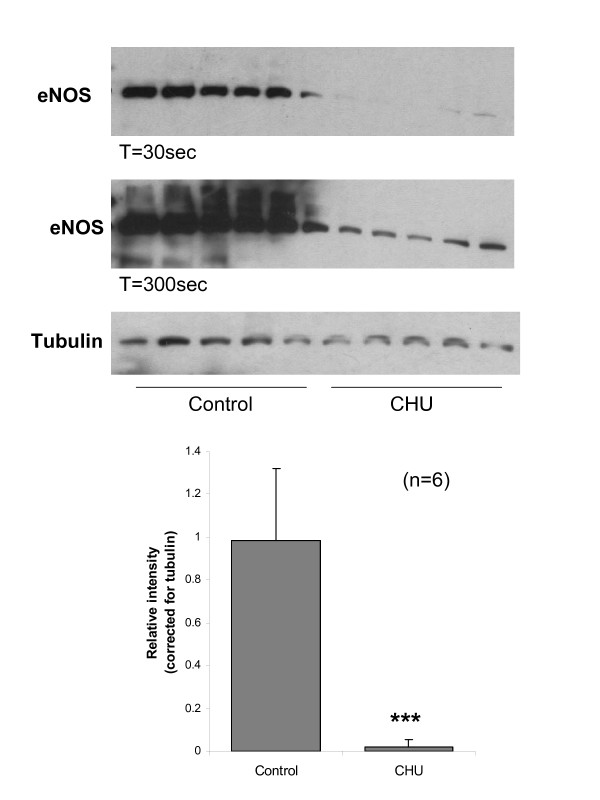
**Protein expression for eNOS and tubulin in whole heart**. Representative autoradiogram taken from control and CHU hearts. Estimation of relative abundance of eNOS and SERCA2 by densitometry. Data represents Mean ± standard deviation of relative expression corrected for tubulin (n = 6 hearts). Statistical comparisons represent: *** P < 0.001. Quantiation made on T = 30 sec autoradiogram to ensure linearity of response for control samples. T = 300 sec autoradiogram included to show presence of eNOS protein at low levels for CHU samples.

**Figure 6:**
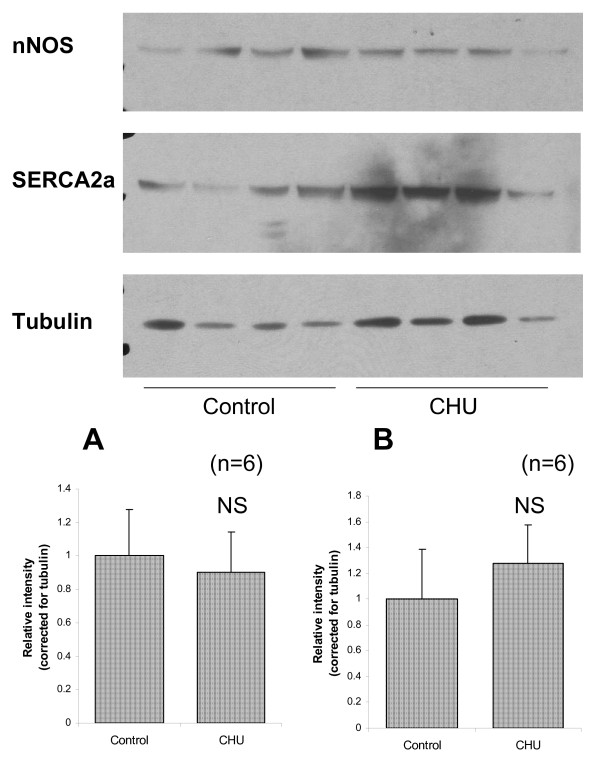
**Protein expression for SERCA2, nNOS and tubulin in whole heart**. Figure represents autoradiogram taken from control and CHU hearts. Data represents mean ± standard deviation for relative densitometric estimates (n = 6 hearts). Statistical significance set at P < 0.05.

Densitometric analysis of Western blot for cardiac tissue revealed a significant decrease in protein corresponding to eNOS for the CHU hearts detectable in the linear range for control samples (Figure 5; P < 0.001). This was verified by both molecular mass and the use of appropriate positive controls (data not shown). Increasing exposure time for autoradiograms revealed that eNOS protein was present at very low levels in CHU rats, however this was beyond the linear range for densitometric estimation. Levels of protein expression for SERCA2 estimated by Western blot were not significantly different for CHU hearts (Figure 6; NS). Levels of protein expression for nNOS were also estimated by Western blot and shown not to be significantly different for CHU hearts (Figure 6; NS). Investigation of iNOS expression revealed this protein was undetectable in control and CHU myocardium (data not shown).

## Discussion

The impact of *in utero *exposure to adverse environmental factors on the development of the fetus is not in doubt, however how these alterations affect myocardium in particular is currently unclear. These experiments represent one of the first detailed investigations of the mechanical performance of the *ex vivo *perfused heart and demonstrate increased contractile performance coupled with impaired relaxation of the heart, in the absence of sympathetic innervation and hormonal inputs. In addition, we demonstrate for the first time that CHU during this critical period of embryonic cardiac development may significantly decrease capillary density for epicardium and endocardium in the adult heart, with possible implications for maintenance of coronary perfusion and cardiac allometric growth.

The increase in body mass for CHU rats is striking, initially observed at weaning and is manifest largely through increased adipose tissue mass. The 2.5-fold increase in relative gonadal WAT mass was similarly distributed in other adipose tissue beds (data not shown), including peri-renal depots, and may reflect a profound metabolic disturbance. Coupled with the apparent decrease in adrenal mass, the CHU rats express a phenotype similar to excessive glucocorticoid treatment [27]. The origins of this are unclear at present, yet enhanced adipose tissue mass coupled with ectopic secretion of glucocorticoid by adipose tissue may result in the adrenal atrophy reported. Consistent with this, CHU in sheep led to increased adipose expression of 11β-hydroxysteroid dehydrogenase-1 enzyme (11β-HSD1), responsible for the tissue-specific recycling of glucocorticoid [28]. Our hypothesis of cardiac hypertrophy following CHU, and previously noted for LV in rats exposed to CHU (days 15–21 of pregnancy [16]) is not proven, however we cannot dismiss the potential in later life. The relative obesity noted may be predisposing factor for cardiac hypertrophy occurring at later stages although none was evident at this young age (9–11 weeks), and is further supported by the preservation of estimated end diastolic volume.

### Cardiac performance

The enhanced cardiac performance for CHU rats observed during early increases in balloon volume (an index of the response to increased preload) may be an early adaptation in contractility to overcome the apparent obesity. However, previous estimates of ventricular volume in rats for this age/cardiac mass [29] suggest that cardiac performance is not significantly different from control when animals are at rest (ventricular volume ~250 μl) and hence the early augmentation in developed pressure (representing volumes of low ventricular filling) with apparently normal cardiac mass may have unrelated origins. To date, we have found no other example of this early increased cardiac performance coupled with preservation of normal performance at higher balloon volumes (higher cardiac work). Overexpression of genes coding for calcium transporters has previously shown to augment contractile performance, including oxerexpression of sarcoplasmic-endoplasmic reticulum Ca^2+ ^ATPase 2 (SERCA2) [30-32], yet overexpression increased cardiac performance at all workloads, rather than over the narrow range recorded here. Normal expression of SERCA2 was confirmed in these hearts, so alternate mechanisms may have been recruited, including alteration to myosin heavy chain sub-type expression.

Impaired relaxation (lusitrophy) has been previously noted for CHU rat hearts, characterised by decreased -dP/dt and increased LVEDP [16] and may be indicative of early fibrosis. This was undetectable using the Masson's Trichrome staining technique used here, however other groups exploiting electron microscopy revealed development of fibrosis at 4 months following CHU [16]. This may represent a methodological discrepancy with Masson's Trichrome being better suited to detection of focal fibrosis rather than diffuse filamentous fibrosis. Previous studies document the influence of nitric oxide on diastolic performance (reviewed [33]) and, notably, in ventricular relaxation in the isolated perfused heart [34]. We cannot rule out the involvement of NO production in the impairment of lusitrophy and the decrease in immuno-detectable eNOS protein may contribute to the impaired production of NO and retard cardiac relaxation in the intact heart [35].

### Capillary density

Early observations suggest that crystalloid-perfused hearts show only ~60% perfusion of the available capillaries for a single-pass [36]. This might imply that substantial decreases in capillary density are possible before impairment of mechanical performance is noted. The decreased capillary density for both epicardium and endocardium for CHU rats is surprising given the preserved cardiac performance and coronary flow, suggesting that oxygenation of the tissue was adequate [37,38]. No measure of arteriolar diameter was possible and dilation is the most likely explanation for preserved coronary flow by means of reduced vascular resistance. Interestingly, these observations raise the possibility of decreased coronary flow reserve in the CHU rat [39], a characteristic previously observed for examples of cardiac hypertrophy [40]. Indeed, over the entire range of balloon volumes studied we show preserved cardiac mechanical performance and coronary flow, implying that significant 'pathological' impairment of coronary flow is necessary to exceed the remaining coronary flow reserve.

Given the preserved coronary flow and sensitive, local control of blood flow to match oxygen delivery with consumption the pathways governing arteriolar dilation, including nitric oxide, may be accentuated. An increase in expression of endothelial nitric oxide synthase (eNOS) as a consequence of decreased capillary density was anticipated given the primary role that endothelial NO release plays in response to increased flow-mediated shear [41] and its induction in response to chronic tissue hypoxia [42]. However, direct measurement by Western blot revealed a decrease in total eNOS protein for CHU hearts, confirming previous observations, but over a different window of hypoxia [12]. Inducible nitric oxide synthase (iNOS) is not normally expressed in the adult heart [43,44] but is induced following ischaemia-reperfusion injury [45] and cardiac failure [46] and was not present at levels detectable by Western blot in the CHU heart. Compensatory expression of nNOS was previously recorded for eNOS-/- mice [47] with specific inhibition of nNOS revealing that flow-induced dilation of coronary vessels was largely controlled by nNOS [48]. However, no compensatory expression of nNOS was noted for CHU rats. Other mechanisms by which the endothelium influences arteriole dilation cannot be ruled out including K_ATP _channels and the prostanoids.

The origins of decreased capillary density for CHU hearts may result following allometric growth [49] of a gestationally small heart [15,50]. Indeed, for control rats capillary density is highest at 1 month of age and falls continually following hypertrophy of the heart as a result of normal growth [51], coupled with increases in myocyte cross-sectional area [52]. Rapid growth in early life may produce a heart of normal mass with relatively fewer cardiomyocytes of larger size [53-55], and poor distribution of capillaries [52].

## Conclusion

We demonstrate that CHU led to enhanced developed pressure and contractility in the isolated perfused heart. Furthermore, we show that this altered performance does not result from changes to SERCA2a protein directly but may result form changes to other Ca^2+ ^pathways. The accompanying decreases in both epicardial and endocardial capillary density may reflect changes to the mechanisms controlling angiogenesis, imposed by the early oxygen deprivation or enhanced allometric growth of a gestationally small heart. In addition, the decreased capillary supply to the myocardium may have consequences for the development of cardiac hypertrophy in adulthood.

## Abbreviations

CHU: Chronic Hypoxia *in utero*; SERCA2a: sarcoplasmic-endoplasmic reticulum Ca^2+ ^ATPase 2a; NOS: nitric oxide synthase; WAT: white adipose tissue; ANOVA: analysis of variance; RPP: rate-pressure product.

## Competing interests

The authors declare that they have no competing interests.

## Authors' contributions

DH designed the studies, undertook the capillary density measurements and undertook the immunoblot analysis. DH also drafted the manuscript and carried out the data analysis and statistical analysis. VO undertook all of the perfusion studies and collected tissue samples. All authors read and approved the final manuscript

## Pre-publication history

The pre-publication history for this paper can be accessed here:


